# Metamorphic Protein Folding Encodes Multiple Anti-*Candida* Mechanisms in XCL1

**DOI:** 10.3390/pathogens10060762

**Published:** 2021-06-17

**Authors:** Acacia F. Dishman, Jie He, Brian F. Volkman, Anna R. Huppler

**Affiliations:** 1 Department of Biochemistry, Medical College of Wisconsin, Milwaukee, WI 53226, USA; adishman@mcw.edu; 2 Medical Scientist Training Program, Medical College of Wisconsin, Milwaukee, WI 53226, USA; 3 Department of Pediatrics, Medical College of Wisconsin, Milwaukee, WI 53226, USA; jhe@mcw.edu

**Keywords:** *Candida*, *C. albicans*, XCL1, metamorphic protein, fold-switching protein, antifungal peptide

## Abstract

*Candida* species cause serious infections requiring prolonged and sometimes toxic therapy. Antimicrobial proteins, such as chemokines, hold great interest as potential additions to the small number of available antifungal drugs. Metamorphic proteins reversibly switch between multiple different folded structures. XCL1 is a metamorphic, antimicrobial chemokine that interconverts between the conserved chemokine fold (an α–β monomer) and an alternate fold (an all-β dimer). Previous work has shown that human XCL1 kills *C. albicans* but has not assessed whether one or both XCL1 folds perform this activity. Here, we use structurally locked engineered XCL1 variants and *Candida* killing assays, adenylate kinase release assays, and propidium iodide uptake assays to demonstrate that both XCL1 folds kill *Candida*, but they do so via different mechanisms. Our results suggest that the alternate fold kills via membrane disruption, consistent with previous work, and the chemokine fold does not. XCL1 fold-switching thus provides a mechanism to regulate the XCL1 mode of antifungal killing, which could protect surrounding tissue from damage associated with fungal membrane disruption and could allow XCL1 to overcome candidal resistance by switching folds. This work provides inspiration for the future design of switchable, multifunctional antifungal therapeutics.

## 1. Introduction

*Candida albicans* and other fungal pathogens cause severe and costly infections in children and adults [1,2,3]. High incidence of drug toxicity and the emergence of resistance limit the utility of available antifungal drug classes [4,5]. Components of the innate immune system termed antimicrobial peptides, including certain chemokines, are currently under investigation as novel antifungal agents [6,7]. Detailed understanding of the relationship between protein structure and function can promote the optimal development of these peptides as therapeutics.

In the field of structural biology, the conventional wisdom has been that each amino acid sequence folds into a single structure to carry out its biological role. However, in recent decades, proteins have been discovered that defy this norm, folding into multiple different structures and reversibly interconverting between them. Recent work has shown that these proteins, called metamorphic proteins, may be more common than initially expected [8,9]. Interest in the biological relevance of metamorphic protein folding is growing, and efforts to understand and harness protein metamorphosis for therapeutic benefit are beginning to mount [10,11,12].

**Figure 1:**
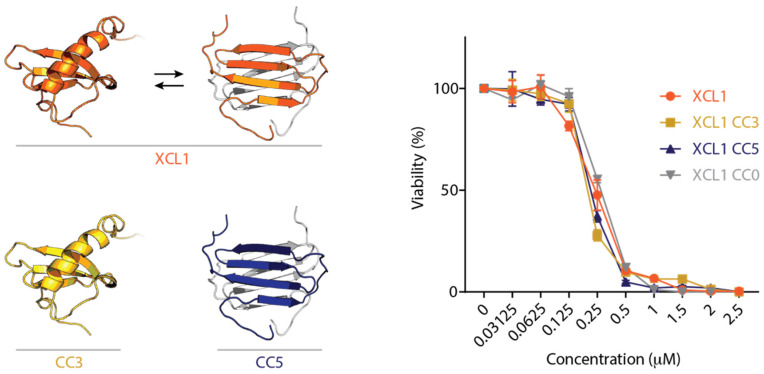
In vitro *C. albicans* killing activity of WT XCL1 and engineered XCL1 variants locked in the chemokine fold, alternate fold, and unfolded state. *Left:* the two XCL1 native structures (chemokine fold, left; dimeric alternate fold, right, in color (subunit A) and light grey (subunit B)). CC3, gold, is an engineered XCL1 variant that is locked in the chemokine fold. CC5, blue, is an engineered XCL1 variant that is locked in the alternate fold. WT XCL1, orange, interconverts between the two folds and occupies each fold in equal proportion under near-physiological conditions. *Right: C. albicans* killing activity of XCL1 (orange), CC3 (gold), CC5 (dark blue), and CC0 (grey). CC0 is an engineered XCL1 variant lacking the disulfide bond that has no defined folded structure.

One such metamorphic protein is the antimicrobial human chemokine XCL1. Chemokines are small, secreted immune proteins that orchestrate the migration of white blood cells under homeostatic and inflammatory conditions, some of which have antimicrobial activity [13,14]. XCL1 is unique amongst chemokines because it switches between the conserved α–β chemokine fold and an all-β alternate fold that forms a dimer [15] (Figure 1). XCL1 occupies the two folds in equal proportion under near-physiological conditions and interconverts between the two folds in the absence of a trigger [15]. The chemokine fold binds and activates XCL1’s cognate G-protein coupled receptor (GPCR), XCR1, on the surface of a subset of dendritic cells [15,16]. The XCL1 alternate fold binds to glycosaminoglycans (GAGs), facilitating the formation of chemotactic concentration gradients [15,17]. It has been shown that the XCL1 alternate fold directly kills *E. coli* via physical membrane disruption, but the XCL1 chemokine fold does not [18,19]. Recent work has demonstrated that wild-type (WT) human XCL1 also kills *C. albicans* [18]. The aim of this study was to determine whether this antifungal activity is encoded by one or both of the XCL1 structures, and to further elucidate the molecular mechanism by which XCL1 kills *Candida*.

Here, we used a panel of engineered XCL1 variants [16,17] and *Candida* killing dose-response and time course assays, adenylate kinase (AK) release assays, and propidium iodide uptake (PI) assays to show that the XCL1 chemokine fold and alternate fold both kill *C. albicans*. However, our data suggest that the two folds kill by different mechanisms. Multiple complementary assays indicate that the alternate fold kills *C. albicans* via direct membrane disruption, in agreement with previous studies [18], but the chemokine fold does not. XCL1 can provide unique therapeutic inspiration as a member of a family of antimicrobial human immune system proteins, with the added feature of switching folds to encode multiple distinct antifungal mechanisms.

## 2. Results

### 2.1. The XCL1 Chemokine Fold, Alternate Fold, and Unfolded State Kill C. albicans

Engineered XCL1 variants have been designed to lock XCL1 into the chemokine fold [16] and the alternate fold [17] by adding a new disulfide bond. The variants are named CC3 (locked chemokine fold) and CC5 (locked alternate fold) (Figure 1). Additionally, an XCL1 variant lacking XCL1’s native disulfide bond, named CC0, has no defined folded structure. We tested WT human XCL1, CC3, CC5, and CC0 for antifungal activity against *C. albicans* using a dose-response plating assay [18,20,21] and found that all of the XCL1 variants kill *C. albicans* with similar dose-response profiles (Figure 1). Previous studies suggest that the XCL1 alternate fold is capable of directly disrupting fungal membranes, but the chemokine fold is not [18]. We thus sought to determine whether the chemokine fold kills via the same mechanism as the alternate fold.

### 2.2. WT XCL1 Kills C. albicans Faster Than an XCL1 Variant Locked in the Chemokine Fold

**Figure 2:**
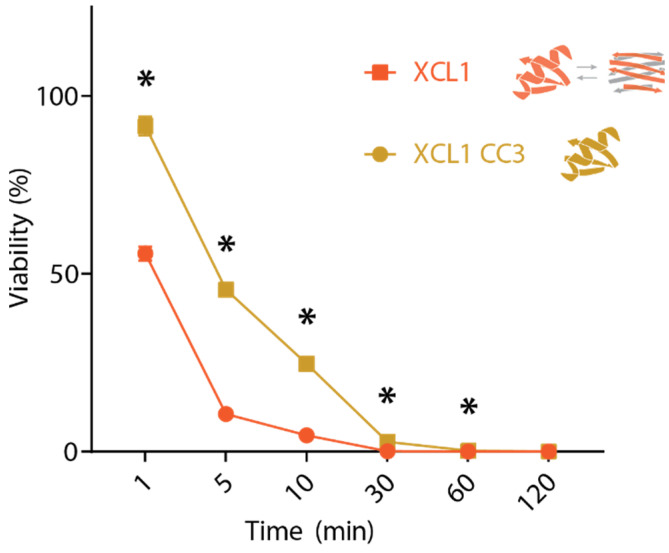
*Candida* killing time course assays for WT XCL1 and CC3. Each timepoint was performed in triplicate. Significant differences between XCL1 and CC3 indicated with * for *p* < 0.05.

If the XCL1 chemokine fold and alternate fold kill *Candida* via the same mechanism, the chemokine fold would be expected to kill with similar kinetics to WT XCL1. We performed time course killing assays for XCL1 and CC3 at a protein concentration of 1 μM against *C. albicans*, finding that CC3 kills more slowly than WT human XCL1 (Figure 2). This difference could occur if the XCL1 alternate fold kills via direct membrane disruption, which occurs quickly, while the XCL1 chemokine fold kills by a slower mechanism. To test the hypothesis that the XCL1 alternate fold kills via membrane disruption while the chemokine fold does not, we performed two complementary assays to assess the ability of the different XCL1 structures to induce membrane disruption in *C. albicans*.

### 2.3. The XCL1 Alternate Fold Induces More Intense Adenylate Kinase Release from C. albicans Than the XCL1 Chemokine Fold

**Figure 3:**
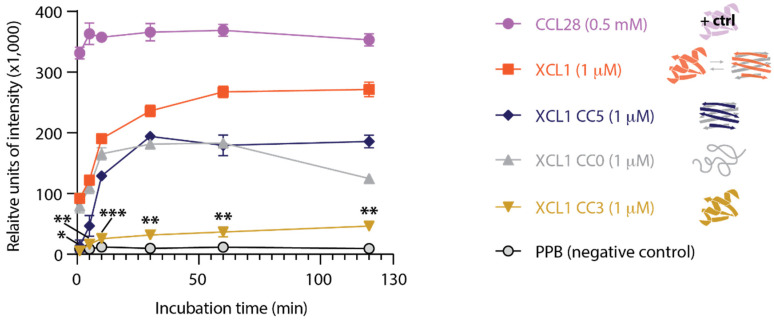
Adenylate kinase (AK) release from *C. albicans* as a measure of membrane disruption in response to XCL1 and structural variants. Each time point was performed in triplicate. The structural behavior of each XCL1 variant is illustrated in the legend. Significant differences between XCL1 and CC3 indicated with * for *p* < 0.0005, ** for *p* < 0.00005, and *** for *p* < 0.000005.

To assess for membrane disruption in *C. albicans* by XCL1 and our panel of locked structural variants, we first performed an adenylate kinase release assay [22,23]. In brief, the dye-based adenylate kinase assay (AKA) measures the release of the intracellular enzyme adenylate kinase (AK) from *C. albicans* cells following protein treatment. As a positive control, we used CCL28, a human chemokine that adopts the conserved chemokine fold and is known to kill *Candida* via direct membrane disruption [24]. The protein suspension buffer, potassium phosphate buffer (PPB), was used as a negative control. We found that XCL1, CC0, and CC5 trigger the release of adenylate kinase from *C. albicans*, suggesting that the XCL1 alternate fold and unfolded state induce *C. albicans* membrane disruption (Figure 3). CC3 treatment resulted in very little adenylate kinase release, even at late time points. CC3 kills with similar potency to XCL1, CC5, and CC0 (Figure 1), but induces less AK release (Figure 3), which suggests that CC3 kills *C. albicans* by a mechanism other than membrane disruption. To confirm these findings, we performed a second complementary assay to detect candidal membrane disruption by XCL1 and CC3.

### 2.4. WT XCL1 Triggers More Propidium Iodide Uptake Than the XCL1 Locked Chemokine Fold Variant

**Figure 4:**
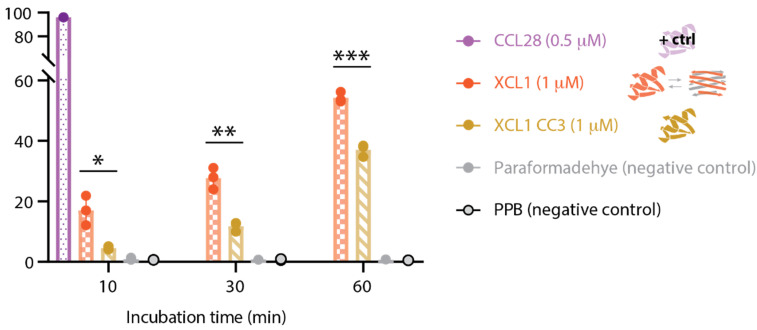
Propidium Iodide uptake by *C. albicans* as a measure of membrane disruption in response to XCL1, CC3, and controls. The timepoint was performed in triplicate. Each data point is represented by a circle. The structural behavior of each variant is illustrated in the legend. Significant differences between XCL1 and CC3 indicated with * for *p* < 0.05, ** for *p* < 0.005, and *** for *p* < 0.0005.

To confirm the finding that WT XCL1 kills *Candida* via membrane disruption but CC3 does not, we performed a propidium iodide (PI) uptake assay, which detects cellular uptake of a dye that selectively labels the chromosome. Higher PI uptake indicates increased membrane disruption. As in the AKA, CCL28 was used as a positive control and PPB was used as a negative control. Paraformaldehyde kills *Candida* but does not induce membrane disruption and was included as a second negative control. We found that WT XCL1 induces significantly higher PI uptake in *C. albicans* than CC3 at 10 min, 30 min, and 60 min after the protein treatment (Figure 4). This suggests that the XCL1 alternate fold kills via membrane disruption, but the XCL1 chemokine fold does not, in concurrence with the AKA data presented in the previous section.

## 3. Discussion

Fungi such as *C. albicans* cause severe infections [1,2,3], but currently available treatments have limited utility due to drug toxicity and the frequent emergence of drug resistance. Thus, new, less toxic antifungal drugs are needed, and antimicrobial proteins in the human immune system, such as chemokines, can inspire the design of such novel therapies [6,7,25,26]. Many human chemokines are known to be antifungal [13,14,21], including the chemokines XCL1 and CCL28 [18]. Indeed, a recent study found that XCL1 and CCL28 are expressed highly in uterine tissue, perhaps suggesting that the antimicrobial properties of these chemokines contribute to the protection of the developing fetus from microbial pathogens [27]. XCL1 has the additional feature of being metamorphic, i.e., switching between multiple different three-dimensional structures reversibly in solution [15]. Interest in metamorphic proteins’ biological and therapeutic utility has grown in recent years [9,10,11,28]. XCL1 can provide unique inspiration for the design of non-toxic, switchable antifungals for which the development of resistance is minimized. Such pursuits rely on understanding structure–function relationships for XCL1 antifungal activity.

The work presented here suggests that XCL1 kills *C. albicans* via two different mechanisms encoded by the two different XCL1 folds. Furthermore, the XCL1 alternate fold appears to kill *Candida* via direct membrane disruption, while the chemokine fold does not. These findings agree with previous work demonstrating that the XCL1 alternate fold induces Negative Gaussian Curvature (NGC), a topological requirement for membrane disruption, in model fungal membranes, while the chemokine fold does not [18].

Inspiration from XCL1 can inform the design of antifungal proteins that switch folds, quickly, reversibly toggling between multiple antifungal killing mechanisms, which would have distinct advantages as antifungal agents. If *Candida* evolves resistance to killing by one protein fold, interconversion to the other fold overcomes this resistance. Additionally, killing by membrane disruption may be advantageous in certain contexts but not others. Killing via a non-membrane-disruptive mechanism may modulate the resulting immune response by decreasing the release of pathogen-associated molecular patterns (PAMPs) and could protect surrounding tissue from damage. Likewise, fold-switching therapies could spare host microbial flora, for example, in the gut, that would be killed by other antifungal agents. Fold-switching proteins could also be designed that have dual functionality, with one fold encoding antifungal activity and the other encoding a distinct therapeutic function. For example, the second fold might encode anti-inflammatory or immunomodulatory activity, reducing the number of drugs required to treat fungal infections. Together, a better understanding of the molecular mechanisms of antifungal action of human metamorphic proteins can inspire the development of improved antifungal therapeutics.

## 4. Materials and Methods

### 4.1. Protein Expression and Purification

Expression of CC0, CC3, CC5, and WT XCL1 was performed as previously described [15,19,29]. In brief, proteins were expressed recombinantly with a His_6_-SUMO tag (pET28a expression vector, BL21 DE3 *E. coli*.). Cultures (grown in terrific broth with 50 mg/mL Kanamycin at 37 °C) were induced with 1 mM isopropyl-b-D-thiogalactopyranoside (IPTG) once they reached an optical density of 0.5–0.7, after which they were grown for an additional 5 h at 37 °C. Cells were harvested by centrifugation (8000× *g*, 10 min) and stored at −80 °C. For protein purification, 50 mM sodium phosphate (pH 8.0), 300 mM sodium chloride, 10 mM imidazole, 0.1% (*v*/*v*) β-mercaptoethanol, and 1 mM phenylmethylsulfonyl fluoride (PMSF) was used to resuspend cell pellets, which were then lysed using a French press or by sonication. Centrifugation was used to collect inclusion bodies from cell lysates (12,000× *g*, 20 min) which were then resuspended and further purified along with the soluble fractions. Nickel column chromatography (nickel resin, Qiagen, Hilden, Germany) was used as an initial purification step. Elution fractions from the nickel columns underwent infinite dilution refolding into 20 mM Tris (pH 8.0), 200 mM sodium chloride, 10 mM cysteine, and 0.5 mM cystine. Refolding solutions were incubated at room temperature with gentle stirring overnight, then concentrated. ULP1 protease cleavage was used to remove the His_6_-SUMO fusion tag, which was then separated from the protein of interest using either cation exchange chromatography (SP Sepharose Fast Flow resin, GE Healthcare, Chicago, IL, USA) or reverse nickel column chromatography (nickel resin, Qiagen). High-performance liquid chromatography was performed with a C18 column as a final purification step. Proteins were then frozen and lyophilized. Sample purities, identities, and homogeneities were checked with matrix-assisted laser desorption ionization time-of-flight (MALDI-TOF) spectroscopy.

### 4.2. Candida Killing Dose-Response Assays

*Candida* killing assays were performed as previously described [18,20,21]. Briefly, a single clone of *C. albicans* strain CAF2-1 was cultured in yeast peptone dextrose (YPD) medium for 16–20 h (30 °C, 250 rpm), before being washed twice and diluted to a final concentration of ~5 × 10^4^ cells/mL in a low-salt, 1 mM potassium phosphate buffer, at pH 7.0 (PPB). Proteins were lyophilized and resuspended to a concentration of 400 μM in 1 mM PPB, aliquoted and stored at −70 °C until use. Proteins were serially diluted with 1 mM PPB and mixed 1:1 with the ~5 × 10^4^ cells/mL *Candida* stocks to a total volume of 200 μL in a 96-well plate. Negative controls were performed using 1 μM PPB. The 96-well plates were incubated for 2 h or the indicated time with gentle shaking (80 rpm). Appropriate dilutions of the suspensions were then plated on YPD agar plates. Plates were incubated at 30 °C for 48 h, then colonies were counted. Viability is reported as the percent colony number with respect to the negative control. Assays were performed in at least duplicate, and protein conditions were tested in triplicate in each assay.

### 4.3. Adenylate Kinase Release Assays

Adenylate kinase leakage was measured with an adenylate kinase assay (AKA) kit (Abcam, MA, USA). *Candida* cells at 2 × 10^7^ CFU/mL in PPB (100 μL) and serial dilutions of CCL28, XCL1, and indicated XCL1 variants (100 μL) were incubated for 30 min in triplicates in 96-well plates. Plates were centrifuged at 3000 rpm for 5 min, and supernatant from each well (100 μL) was transferred to the white 96-well plate and mixed with adenylate reagent (100 μL) in white 96-well flat-bottom assay plates. Luminescence was measured immediately by a SpectraMax plate reader (Molecular Devices, San Jose, CA, USA).

### 4.4. Propidium Iodide Uptake Assays

*Candida* was cultured and washed as described above. *Candida* suspensions at 5 × 10^6^ CFU/mL were incubated with equal volumes of 1 mM PPB, CCL28, XCL1, or XCL1 CC3 solutions (final concentration 0.5 μM), or paraformaldehyde solution (2% paraformaldehyde, 0.5% bovine serum albumin (BSA), and 2 mM EDTA in PBS) at room temperature for 10, 30 and 60 min in triplicates. Cells were washed and resuspended with 1× FACS (PBS with 2% fetal bovine serum and 2 mM EDTA buffer) and then stained with propidium iodide (PI; Thermo Fisher Scientific, Grand Island, NY, USA) at 1 mg/mL at room temperature in the dark for 10 min. Stained cells were washed with 1× PBS and resuspended in 200 μL of the paraformaldehyde solution. Cellular PI uptake was measured by flow cytometry in the CRI flow cytometry core on a BD LSR II (BD Biosciences, San Jose, CA, USA) and analyzed with FlowJo (Tree Star, Inc., Ashland, OR, USA).

## Data Availability

The data presented in this study are available within the article.
